# Functional Capacity in Patients Who Recovered from Mild COVID-19 with Exertional Dyspnea

**DOI:** 10.3390/jpm12060874

**Published:** 2022-05-26

**Authors:** Yaniv Dotan, Elite Weiner, Merav Zucker-Toledano, Anna Solomonov, Eyal Fuchs, Hanna Dawood, Elad Mor, Moneera Hanna, Rihan Naser-Aldeen, Lea Bentur, Ronen Bar-Yoseph

**Affiliations:** 1 Division of Pulmonary Medicine, Rambam Health Care Center, Technion Faculty of Medicine, Haifa 3200003, Israel; y_dotan@rambam.health.gov.il (Y.D.); a_solomonov@rambam.health.gov.il (A.S.); 2 Division of Pulmonary Medicine, Rambam Health Care Center, Haifa 3109601, Israel; e_weiner@rambam.health.gov.il (E.W.); e_fuchs@rambam.health.gov.il (E.F.); h_dawood@rambam.health.gov.il (H.D.); e_mor@rambam.health.gov.il (E.M.); 3 Pediatric Cardiology Institute, Ruth Children’s Hospital, Rambam Health Care Center, Haifa 3109601, Israel; meravzt8@gmail.com; 4 Pediatric Pulmonary Institute, Ruth Children’s Hospital, Rambam Health Care Center, Haifa 3109601, Israel; m_hanna@rambam.health.gov.il; 5 Technion Faculty of Medicine, Haifa 3109601, Israel; rihan@campus.technion.ac.il; 6 Pediatric Pulmonary Institute, Ruth Children’s Hospital, Rambam Health Care Center, Technion Faculty of Medicine, Haifa 3200003, Israel; r_bar-yoseph@rambam.health.gov.il

**Keywords:** mild COVID-19 disease, cardiopulmonary exercise test, exertional dyspnea

## Abstract

Background: Post mild COVID-19 dyspnea is poorly understood. We assessed physiologic limitations in these patients. Methods: Patients with post mild COVID-19 dyspnea (group A) were compared (pulmonary function tests, 6-min walk test (6MWT), echocardiography and cardiopulmonary exercise test (CPET)) to post moderate/severe COVID-19 (group B) and to CPET and spirometry of patients with unexplained dyspnea (group C). Results: The study included 36 patients (13 in A, 9 in B and 14 in C). Diffusion capacity was lower in group B compared to group A (64 ± 8 vs. 85 ± 9% predicted, *p* = 0.014). 6MWT was normal and similar in both patient groups. Oxygen uptake was higher in group A compared to groups B and C (108 ± 14 vs. 92 ± 13 and 91 ± 23% predicted, *p* = 0.013, 0.03, respectively). O_2_ pulse was normal in all three groups but significantly higher in the mild group compared to the control group. Breathing reserve was low/borderline in 2/13 patients in the mild group, 2/9 in the moderate/severe group and 3/14 in the control group (NS). Conclusions: Patients with post mild COVID-19 dyspnea had normal CPET, similar to patients with unexplained dyspnea. Other mechanisms should be investigated and the added value of CPET to patients with post mild COVID-19 dyspnea is questionable.

## 1. Introduction

Infection with severe acute respiratory syndrome coronavirus-2 (SARS-CoV-2) leads to severe disease called coronavirus disease 2019 (COVID-19) requiring hospitalization in up to 20% of cases [1]. Respiratory manifestations from SARS-CoV-2 infection can range from mild pneumonia to life-threatening hypoxia secondary to severe acute respiratory distress syndrome (ARDS) and mechanical ventilation (MV) in about 12–24% of hospitalized patients [2,3]. Risk factors for development of severe disease include (in order of magnitude) age ≥ 65 years, immunosuppression, pulmonary disease, liver disease, chronic kidney disease, neurologic disease, diabetes and cardiac disease [4]. Autopsy reports describe pulmonary findings of diffuse alveolar damage and micro-vessel thrombosis in patients who died of COVID-19-related ARDS [5,6]. There appears to be markedly increased inflammatory response of the lungs to SARS-CoV-2 with increased systemic inflammatory markers and high concentrations of SARS-CoV-2 RNA in the lungs [7]. Respiratory recovery is usually slow, with most patients on MV for at least a couple of weeks [8]. The first reports regarding patients who recovered from severe COVID-19 demonstrated that the vast majority (94%) had residual disease on chest computed tomography (CT) with ground-glass opacities (GGO) as the most common pattern [9]. This and previous reports regarding survivors of other coronavirus pneumonias (severe acute respiratory syndrome (SARS) and Middle East respiratory syndrome (MERS)) showing persistent impairments of pulmonary function raised the concern that COVID-19 might also damage the lungs chronically [10,11,12,13]. In studies in SARS survivors, there was persistent and significant impairment of exercise capacity and health status over 24 months based on pulmonary function tests (PFTs), 6-min walk test (6MWT) and health questionnaire, and 40% of the survivors still had symptoms of chronic fatigue syndrome (CFS) after 3.5 years [12,14].

In the longer term, there is a significant concern that severe COVID-19 can lead to organizing pneumonia with evolution to widespread fibrotic changes as seen in fatal cases of COVID-19 at autopsy [15]. Recovery from COVID-19 usually takes 2–6 weeks but, in some patients, symptoms such as fatigue, loss of taste or smell, headache, body aches, diarrhea, nausea, and general weakness may linger for months after initial recovery [16]. These symptoms were initially termed post-acute COVID-19 syndrome if persisting beyond four weeks after the beginning of the acute disease [17]. If these symptoms persist beyond three months, this syndrome has a variety of names, including long COVID or long-haul COVID, and is listed in the ICD-10 classification as post-COVID-19 condition (PC19C) since September 2020. Any patient with COVID-19 may develop PC19C regardless of the severity of the initial infection [18]. A wide range of causes relating to PC19C have been identified, including organ damage, chronic inflammation, non-specific effects of hospitalization, critical illness and post-intensive care syndrome, persistent viremia and complications related to underlying long-term conditions or to treatments used during episodes leading to adverse drug reactions [19,20]. A few studies have evaluated patients, mainly post-hospitalization with severe COVID-19, with cardiopulmonary exercise tests (CPETs) with conflicting results [21,22,23,24,25]. However, in most cases of PC19C, the etiology of dyspnea is unknown, especially in mild cases. CPET measures gas exchange parameters as well as other physiological parameters, such as respiratory rate, tidal volume, blood pressure, oxygen saturation, 12 lead ECG and pulmonary function tests from rest to peak exercise and post-exercise response. Hence, CPET provides a comprehensive assessment of the exercise response, and reflects the influences and interactions of the cardiac, respiratory, musculoskeletal and hematological systems. Integration of the physiologic data allows an analysis of the system as a whole, while separate analyses help determine which system(s) limit exercise capacity or are related to exertional dyspnea.

Since there are no obvious mechanisms explaining prolonged respiratory symptoms in patients who recovered from mild COVID-19 (without pulmonary involvement by definition) to date, we sought to focus on patients who had mild COVID-19 who complained of breathless and had normal PFTs and echocardiography. Our objectives were to demonstrate if there are objective physiologic limitations by CPET, and if so, to investigate whether the limitations are caused by pulmonary, cardiac, or other physiological factors by CPET (e.g., low fitness). We believe that revealing mechanisms for breathless in this vast group of patients might allow relief and reassurance for these patients and might potentially serve as a nidus towards specific remedies.

## 2. Methods

### 2.1. Research Design

This was a prospective observational study of a cohort of patients who recovered from COVID-19 compared to a cohort of patients with unexplained dyspnea (subjective complaint of dyspnea with normal physical examination, normal pulmonary function test, no known respiratory or cardiac pathology) who underwent CPET in our institution. We assessed COVID-19 severity by the World Health Organization (WHO) definition [26]:

1. Mild disease–symptomatic patients without evidence of viral pneumonia or hypoxia.
2. Moderate disease–patients with clinical signs of pneumonia but no signs of severe pneumonia, including oxygen saturation (SpO_2_) ≥ 90% on room air.
3. Severe disease–severe pneumonia: clinical signs of pneumonia plus one of the following: respiratory rate > 30 breaths/min; severe respiratory distress; or SpO_2_ < 90% on room air.
4. Critical disease–COVID-19 related ARDS.

### 2.2. Patients

Patients included in the study were adult patients recovering from COVID-19 with a documented infection with SARS-CoV-2, breathless more than 3 months after the acute disease, cognitive ability to sign informed consent and physical ability to participate in exercise tests. Patients with severe pulmonary or cardiac disease prior to COVID-19, pregnant women, and patients with active infection or cancer were excluded. A cohort of patients matched by age and BMI who underwent CPET due to unexplained dyspnea was used as a comparison to the study patients. In the control group were included only patients who completed the CPET and in whom no obvious etiology (i.e., cardiac, pulmonary) was found after interpretation of the test.

This study was conducted in accordance with the amended Declaration of Helsinki. Rambam Heath Care Campus review board approved the protocol, and written informed consent was obtained from all patients (protocol number 0760-20-RMB, 0106-17-RMB). Clinical trial registration number: NCT05323760.

### 2.3. Study Protocol

Patients who had COVID-19 with ongoing prolonged symptoms were invited for clinical evaluation to the post-COVID clinic in the Pulmonary Division of Rambam Health Care Campus, Haifa, Israel. From this post-COVID clinic, patients were screened for the study. Those who fulfilled the inclusion and exclusion criteria, and agreed to participate in the study, signed informed consent and were recruited. PFTs were performed in accordance with the ATS/ERS (American Thoracic Society/European Respiratory Society) Task Force, using a Quark PFTs spirometer (Cosmed, Rome, Italy). All patients had a clinical exam and full set of PFTs, including spirometry (forced expiratory volume in the first second (FEV_1_), forced vital capacity (FVC)), volumes (total lung capacity (TLC), residual volume (RV)) and diffusion capacity of the lungs to carbon monoxide (DLCO) measurements per guidelines [27,28].

Maximal voluntary ventilation (MVV), also referred to as maximal breathing capacity, was defined as the maximum volume of ventilation achieved in one minute. Subjects were instructed to breathe rapidly and deeply, ventilatory volumes were recorded, and the maximal volume achieved over 12–15 consecutive seconds was expressed in liters per minute. Spirometry was completed again before the CPET and 10 min’ post-exercise, with a fall of >12% in FEV_1_ considered as exercise-induced bronchoconstriction.

Participants were referred to echocardiography, 6MWT and CPET. Echocardiography was carried out and evaluated according to international guidelines and optimally done prior to the CPET [29]. 6MWT was performed according to the guidelines of the ATS [30]: The walk was performed indoors, along a 30-m-long flat and straight corridor, marked every 3 m, and turnaround points marked with a cone. The patients were instructed to walk as far as possible for 6 min [30].

CPET was carried out by an experienced technician (HM) and a pediatric pulmonologist (RBY) using a Quark CPET metabolic cart (Cosmed, Rome, Italy) according to ATS guidelines [31] and conducted on a treadmill with Bruce Ramp protocol after 2 min of rest and 1 min of warm-up, with incremental speed and slope until exhaustion. Gas exchange variables through a designated face mask (V2 mask, Cosmed, Rome, Italy), 12-lead electrocardiogram (ECG), blood pressure and SpO_2_ were recorded at rest, during exercise and in the recovery period. SpO_2_ was measured continuously using Masimo SET 2000 (Schiller, Washington, DC, USA) and recorded at baseline, every 120 s, peak exercise and one, two- and five-minutes post-exercise. Criteria for terminating the test were inability to continue walking/running in association with subjective evidence of fatigue (sweating, hyperpnea), and one or more of the following: peak VO_2_ > 80% predicted, maximal heart rate > 80% HR predicted (HRpred = 208 − (Age × 0.7)) [32], RER > 1.05, or reaching a VO_2_ plateau (failure to increase oxygen uptake despite a continues increase in work). Breathing reserve (BR) was calculated as (MVV − peak minute ventilation (VE) and (1 − peakVE/MVV) × 100) and low BR defined as BR% < 15% or BR < 11 L/min [33]. Patients with normal BR were compared to patients with borderline/low BR regardless of their group (mild, moderate/severe and control groups) in order to understand whether prediction of patients with low/borderline BR was possible based on initial tests (i.e., PFTs) before referring to CPET.

### 2.4. Statistical Analysis

Data are presented as mean ± SD for continuous variables or percentages for categorical variables. Chi-squared was used for categorical data, and the paired or unpaired t-test was used for continuous data. Fisher’s exact test was used for simple between-group comparisons. Missing data were considered missing at random and handled by omitting the cases with the missing data and analyzing the remaining data for the specific analysis. *p* values < 0.05 were considered statistically significant. All data were analyzed using SPSS (version 12.0; SPSS Inc., Chicago, IL, USA).

This study was conducted in accordance with the amended Declaration of Helsinki. Rambam Heath Care Campus review board approved the protocol, and written informed consent was obtained from all patients (protocol number 0760-20-RMB).

## 3. Results

### 3.1. Patients

**Figure 1:**
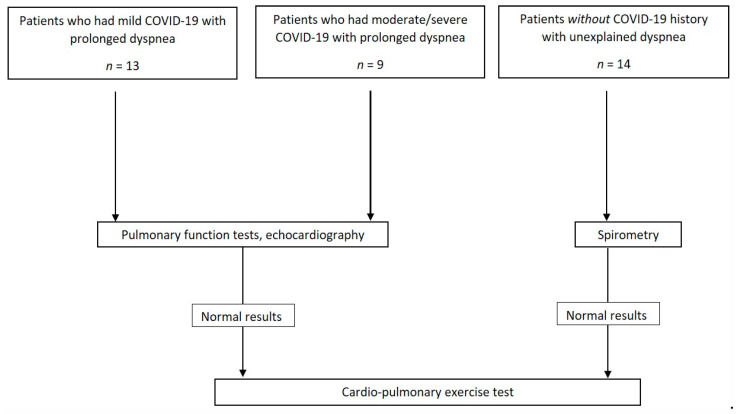
Flow chart of participants in the study.

The study included a total of 22 patients who recovered from acute COVID-19 and had prolonged symptoms, including breathless, and a control group of 14 patients with unexplained dyspnea who underwent CPET (Figure 1). Of the 22 COVID-19 patients, none were vaccinated with COVID-19 vaccine prior to their acute disease, 13 had mild disease and were not hospitalized during their acute disease, and 9 had moderate (*n* = 2) or severe (*n* = 7) disease (see WHO definitions for disease severity in Methods section); all of them were hospitalized for an average of 7 ± 5 days. The mild disease group was significantly younger than the moderate/severe group (29 ± 16 vs. 53 ± 4 years, *p* = 0.035) and had similar ages to the control group (32 ± 10 years, *p* = 0.42). BMI was elevated and similar in all groups (29 ± 7 vs. 31 ± 3 vs. 27 ± 6 kg/m^2^, respectively). All patients with mild disease did not have documented pulmonary involvement and did not require oxygen in their acute phase of disease. All patients with moderate/severe disease had pneumonia, but only patients with severe disease required oxygen during their hospitalization. All patients (COVID-19 and control) were generally healthy, and none had prior significant heart or lung diseases. One patient from the mild group had hypertension before COVID-19. From the moderate/severe group, three had hypertension, two had diabetes mellitus, and one had a history of cancer (ovarian). Patients in the unexplained dyspnea group were healthy without a significant previous medical history. Demographic and baseline data are presented in Table 1.

### 3.2. PFTs, 6MWT and Echocardiography

All patients in the mild and moderate/severe COVID-19 groups had a full set of PFTs, including spirometry (FEV_1_, FVC), volumes (TLC, RV), and diffusion (DLCO) measurements. Patients in the control group had only spirometry measurements. Spirometry values were normal in all three groups. Volumes were not statistically significantly different between the mild and moderate/severe groups. Diffusion capacity (DLCO) was significantly lower in the moderate/severe group compared to the mild group (85 ± 9 vs. 64 ± 8% of predicted, *p* = 0.014). Walking distance in 6MWT was normal and similar in both mild and moderate/severe groups (594 ± 128 vs. 593 ± 89 m, *p* = 0.98). All patients in all three groups had normal echocardiography except for one patient in the mild group who had grade 1 diastolic dysfunction. Table 2 summarizes PFTs and 6MWT data.

### 3.3. CPET Results

**Figure 2:**
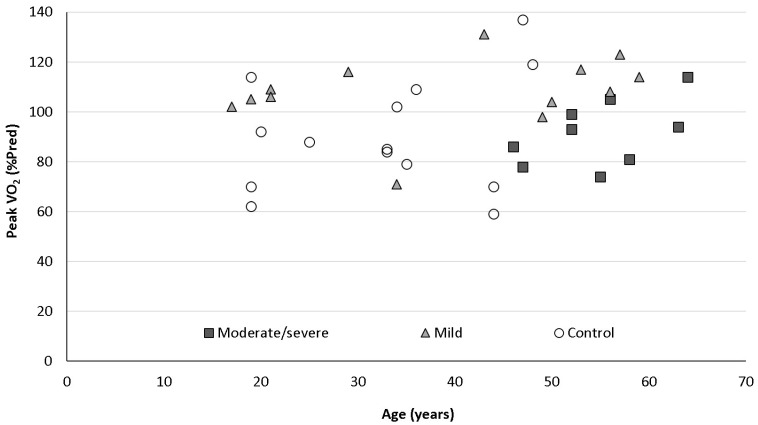
Peak oxygen uptake (peakVO_2_%pred) and age relationship for the 3 groups. Mild patients (gray triangles) had higher peakVO_2_ than the moderate/severe patients (black squares) and the control group (open circles).

All patients had CPET as described in the Methods section. All value averages for all three groups were within normal limits, including peak oxygen uptake (peak VO_2_), heart rate, ventilatory equivalents (VE/VCO_2_ slope), O_2_ pulse (VO_2_/HR), saturation before and at peak exercise, VE, MVV and BR. BR was low/borderline in 2/13 (15%) patients in the mild group, 2/9 (22%) in the moderate/severe group and 3/14 (21%) patients in the control group (NS for all between groups comparisons). Peak VO_2_% of predicted was normal in all groups but was significantly higher in the mild group compared to the moderate/severe and control groups (108 ± 14 vs. 92 ± 13 and 91 ± 23 %pred, *p* = 0.013, 0.03, respectively). Absolute peak VO_2_ was normal in the mild COVID-19 and control groups and borderline and significantly lower in the moderate/severe COVID-19 group (33 ± 9.9, 35 ± 10 and 23 ± 2.7 mL/kg/min, respectively, *p* = 0.008 for comparison between mild vs. moderate/severe groups). O_2_ pulse was normal in all three groups but significantly higher in the mild group compared to the control group (101 ± 29 vs. 93 ± 26 %pred, *p* = 0.046). When dividing all patients included in the study (COVID-19 and control, *n* = 36) to normal (*n* = 29) and low/borderline (*n* = 7) BR, the latter group had significantly lower FEV_1_ and FVC (97 ± 11 vs. 78 ± 13 and 97 ± 14 vs. 80 ± 16 %pred, *p* = 0.02, 0.024, respectively) and higher O_2_ pulse (93 ± 25 vs. 108 ± 13 %pred). Patients with low/borderline BR were older, more males than females and more obese, but none were statistically significant. Table 3 and Figure 2 summarize the CPET data for all three groups (mild, moderate/severe COVID-19 and control groups). Table 4 summarizes the CPET data by BR (normal and low/borderline).

## 4. Discussion

In the current study, we evaluated patients who had recovered from mild COVID-19 complaining of breathless and compared them to patients who recovered from moderate/severe COVID-19 and to patients with unexplained dyspnea. Patients who recovered from mild COVID-19 with dyspnea did not have physiologic limitation on CPET, while patients who recovered from moderate/severe COVID-19 had lower diffusion capacity (DLCO) and lower peak VO_2_ compared to patients who recovered from mild disease and patients with unexplained dyspnea. Hence, dyspnea in patients who recovered from mild COVID-19 without pulmonary involvement during the acute phase of the disease did not have a physiologic explanation in our study and the added value of CPET after PFTs in patients recovering from mild COVID-19 is questionable.

The first reports regarding patients who recovered from severe COVID-19 demonstrated that the vast majority (94%) had residual disease on chest CT with GGO the most common pattern [9]. At 30 days after discharge due to COVID-19 pneumonia, about half the patients had abnormal chest CT findings and about three-quarters had abnormal PFTs with DLCO the most frequent abnormal value [34]. Long term dyspnea is common following hospitalization due to COVID-19: of 538 survivors who were hospitalized, approximately 40% still reported dyspnea three months after the acute illness [35]. Of 100 patients who were discharged from ICU (*n* = 32) or a general ward (*n* = 68), dyspnea was reported in 65.6% and 42.6%, respectively [36]. A study that included 1816 patients who were hospitalized due to COVID-19 showed that fatigue, dyspnea, chest pain, and cough were the most prevalent respiratory symptoms found in 52%, 37%, 16% and 14% of patients between three weeks and three months, respectively [37]. In a long term follow-up of 1276 hospitalized COVID-19 survivors, a quarter of the patients still reported dyspnea at six months and 30% at one year after the acute illness [38].

Few studies have evaluated patients recovering from COVID-19 with CPET. The largest study included 200 patients three months after hospitalization due to COVID-19. About half had lower than predicted peak VO_2_; among them 14.8% had respiratory, 34.4% had cardiac, and 50.8% had non-cardiopulmonary reasons for exercise limitation [22]. However, this study did not include patients with mild disease who were not hospitalized. Another large study evaluated patients after acute COVID-19 with and without PC19C with CPET at three months after the acute disease. Patients with PC19C had significantly lower peak VO_2_ compared to asymptomatic subjects, developed symptoms more frequently during CPET and were less likely to reach the anaerobic threshold when compared to asymptomatic subjects [24]. In this study, about a fifth of the patients required hospitalization in their acute phase of COVID-19. Another study compared 38 patients who recovered from COVID-19 and were not hospitalized to 25 patients who were hospitalized due to COVID-19. The most common symptom in both groups was fatigue followed by exertional dyspnea. Patients who were hospitalized had lower FVC, TLC and DLCO and lower peak VO_2._ Interestingly, 68% of hospitalized patients had chronotropic insufficiency compared to 18% of patients who were not hospitalized [39].

In our study, we compared three groups of patients: patients recovered from mild COVID-19, patients recovered from moderate/severe COVID-19, and a control group with unexplained dyspnea. Patients with mild disease had normal PFTs at three months after the acute illness. Patients with moderate/severe disease had normal spirometry and volumes but abnormal and significantly lower DLCO compared to patients with mild disease. Patients who recovered from moderate/severe COVID-19 complaining of dyspnea had lower peak VO_2_ than patients who recovered from mild disease and patients with unexplained dyspnea. Interestingly, patients with mild disease had, on average, significantly higher peak VO_2_ and O_2_ pulse (VO_2_/HR, low value or flattening of the O_2_ pulse curve are considered markers for decreased cardiac stroke volume), despite reporting dyspnea.

Exertional dyspnea was assessed using ventilatory responses, such as high minute ventilation (VE), breathing reserve (BR), ventilatory equivalents (VE/VCO_2_ slope, a submaximal marker for increased ventilatory drive related to the amount and sensitivity of central chemoreceptors and the ventilatory dead space), oxygen saturation and exertional cardiac parameters (especially O_2_ pulse). No differences were found in the above parameters between the mild and control groups. When we divided all patients into two groups by normal or low/borderline BR, we found that patients with low/borderline BR had pretest (CPET) abnormal PFTs, explaining and predicting the respiratory limitation seen in patients who suffered physical lung damage.

Exertional dyspnea may also be related to obesity and deconditioning. In our study, most of the patients were overweight/obese but with normal mean oxygen uptake and similar to the overweight control group.

We cannot explain physiologically the dyspnea seen in patients who recovered from COVID-19 without pulmonary/cardiac dysfunction, illustrated by normal PFTs/echocardiography, respectively. Possible mechanisms that should be considered include venous thromboembolic disease (VTE), deconditioning, pulmonary/cardiac dysfunction not discovered in routine tests, hypothyroidism or other endocrine dysfunction, depression/anxiety related or a chronic fatigue syndrome-like clinical picture. In two meta-analyses, it was shown that the prevalence of VTE in patients with COVID-19 is very high; 26% among about 3500 patients, 32% pulmonary embolism (PE) and 27% deep vein thrombosis (DVT) among 6500 patients [40,41]. However, these patients were hospitalized mostly with severe or critical illness and there is no available data regarding the prevalence of VTE in patients who had mild disease and were not hospitalized. Deconditioning is a plausible explanation, but one would expect lower anaerobic thresholds, lower maximal oxygen consumption and lower workload, which we did not see in our study. Pulmonary and/or cardiac dysfunction not demonstrated on routine tests (PFTs/echocardiography) would be expected to be revealed in CPET, which was not seen here. Moreover, patients with mild disease had significantly higher O_2_ pulse, representing theoretically higher stroke volumes, and did not show abnormal heart rate response.

Regarding cardiac damage that cannot be demonstrated in routine tests, in a cohort of 100 patients who recovered from COVID-19, 78% had cardiovascular involvement as detected by standardized cardiac MRI, irrespective of pre-existing conditions, the severity and overall course of the COVID-19 presentation, the time from the original diagnosis, or the presence of cardiac symptoms [42]. On the other hand, among 41 patients with dyspnea who had mild COVID-19 and were not hospitalized and 42 control participants, parameters indicating myocardial inflammation and edema were comparable between patients and control with no visible myocardial edema in any of the participants [43].

PC19C is frequently associated with continuing respiratory problems and debilitating fatigue of which patients can interpret and present with dyspnea. From our clinical experience in a busy post-COVID-19 clinic, many patients with PC19C report persistent symptoms that resemble CFS. In a small study, CFS-like features were found in 27% of patients who recovered from COVID-19. These patients showed worse sleep quality, fatigue, pain, depressive symptoms, subjective cognitive complaints, and dyspnea [44]. CFS is linked to a viral and autoimmune pathogenesis. The underlying pathophysiology involves both initiation or trigger by viral disease and, in a significant subset, an autoimmune etiology. There is now increasing evidence that a great variety of autoantibodies may be driving severe forms of COVID-19. These autoantibodies may also play a crucial role in the extended multi-organ illness persevering for months in patients with PC19C [45]. The symptoms that occur in a large number of patients following severe COVID-19 disease, but even in mild cases, are similar to the clinical symptoms of other forms of infection-triggered CFS. However, we did not evaluate methodologically the patients’ mental/psychological state and, therefore, cannot refer to this theory in our patients robustly.

Patients with PC19C resemble clinically patients with hypothyroidism and/or adrenal insufficiency. A recent prospective observational study found abnormal thyroid function tests in about 15% of patients after hospitalization due to COVID-19, suggesting that SARS-CoV-2 might directly induce viral thyroiditis [46]. Most patients with severe COVID-19 disease receive dexamethasone and may have an adrenal pre-damaged by the inflammatory process [47]. Therefore, there may be a predisposition for adrenal insufficiency explaining some of the symptoms in patients with PC19C [48]. However, both theories are preliminary and not seen widely clinically in patients with PC19C, and were not examined systemically in our study. Lastly and interestingly, a novel study on 10 patients with breathless 1 year after mild COVID-19 who underwent invasive CPET (including right heart catheterization and arterial line) showed a peripheral, rather than a central (cardiac) limitation to exercise. The limit was characterized by diffusion defect in oxygen delivery (impaired systemic oxygen extraction) in patients who have recovered from COVID-19 demonstrating a depressed aerobic exercise capacity suggesting impaired oxygen extraction, which was attributed primarily to reduced oxygen diffusion in the peripheral microcirculation [49].

Our study has several limitations. This is a single-center study; the sample size is small with inherently reduced statistical power. However, it is relatively large for a complex physiologic study carried out during an epidemic. CT scans of the mild patients were not performed to rule out some unexpected GGO changes. No respiratory or quality of life questionnaires were completed.

In summary, patients who recovered from mild COVID-19 complaining of dyspnea had normal PFTs and normal echocardiography. Despite dyspnea, physiological abnormalities on CPET were similar to matched comparators referred for unexplained dyspnea without a history of COVID-19. There is currently no known obvious reason why this group of patients reported dyspnea as opposed to patients who recovered from COVID-19 that involved the lungs that had lower diffusion capacity in our study. Other mechanisms, such as a CFS-like clinical syndrome related to SARS-CoV-2 should be investigated. 

## Figures and Tables

**Table 1:** Demographic and baseline data for all three groups: mild COVID-19, moderate/severe COVID-19 and control groups (severity refers to the acute phase of the disease, see Methods section).

	Mild (*n* = 13)	Moderate/Severe (*n* = 9)	Control Group (*n* = 14)
Age (years)	37 ± 16 *	53 ± 4	33 ± 11
Gender (%F)	62%	45%	29%
BMI (kg/m^2^)	29 ± 7	31 ± 3	27 ± 6
Hospitalization (days)	0	7 ± 5	NR
Supplemental oxygen (% needed)	0	78%	NR
Lung disease (%)	0	0	0
Heart disease (%)	0	0	0
Hypertension (%)	8%	33%	0
Diabetes mellitus (%)	0	22%	0
History of cancer (%)	0	11%	0
Active smoker (%)	8%	22%	21%

* Statistically significant between mild vs. moderate/severe groups; BMI—body mass index; NR—not relevant.

**Table 2:** PFTs and 6MWT data for all 3 groups: mild, moderate/severe and control groups.

	Mild (*n* = 13)	Moderate/Severe (*n* = 9)	Control Group (*n* = 14)
FEV_1_ (% of predicted)	98 ± 13%	85 ± 17%	95 ± 11%
FVC (% of predicted)	99 ± 18	81 ± 16	97 ± 10
FEV_1_/FVC	0.82 ± 0.08	0.83 ± 0.03	0.82 ± 0.06
TLC (% of predicted)	88 ± 11	88 ± 9	NA
RV (% of predicted)	100 ± 28	114 ± 13	NA
DLCO (% of predicted)	85 ± 9 *	64 ± 8	NA
6MWD (meter)	594 ± 128	593 ± 89	NA

* Statistically significant between mild vs. moderate/severe groups; PFTs—pulmonary function tests; 6MWT—six-minute walk test; FEV_1_—forced expiratory volume in the first second; FVC—forced vital capacity; TLC—total lung capacity; RV—residual volume; DLCO—diffusion capacity of the lung to carbon monoxide; 6MWD—6-min walk distance; NA—not available.

**Table 3:** CPET data for all 3 groups: mild, moderate/severe COVID-19 and control groups.

	Mild (*n* = 13)	Moderate/Severe (*n* = 9)	Control Group (*n* = 14)
PeakVO_2_ (ml/kg/min)	33 ± 9.9 *	23 ± 2.7	35 ± 10
PeakVO_2_ (%pred)	108 ± 14 ᶲ	92 ± 13	91 ± 23
RER	1.15 ± 0.17	1.18 ± 0.18	1.24 ± 0.1
PeakHR (bpm)	177 ± 19	164 ± 18	183 ± 13
PeakHR (%pred)	100 ± 6	96 ± 11	99 ± 6.2
VE/VCO_2_ slope	31 ± 7.7	34 ± 6.6	29 ± 6.4
PeakO_2_ pulse (%pred)	101 ± 29 ^	95 ± 12.3	93 ± 26
SpO_2_ pretest (%)	98 ± 1.5	99 ± 1	99 ± 1
Peak SpO_2_ (%)	98 ± 1.3	96 ± 4	98 ± 1.6
VE (L/min)	108 ± 31	87 ± 18	115 ± 33
MVV (L/min)	133 ± 31	113 ± 28	124 ± 42
BR (L)	27 ± 12.6	26 ± 13	42 ± 30
BR (%)	20 ± 9.5	21 ± 11.3	27 ± 18

* Statistically significant between mild to moderate/severe group; ᶲ—Statistically significant between mild to moderate/severe and control groups; ^—Statistically significant between mild and control groups; CPET—cardiopulmonary exercise testing; PeakVO_2_—peak oxygen uptake; RER—respiratory exchange ratio; HR—heart rate; VE/VCO_2_—minute ventilation/carbon dioxide production; SpO_2_—oxygen saturation; Peak SpO_2_—oxygen saturation at peak exercise; MVV—maximal voluntary ventilation; BR—breathing reserve.

**Table 4:** CPET data by division to normal and low/borderline breathing reserve for all patients (mild, moderate/severe COVID-19 and control groups).

	Normal BR (*n* = 29)	Low/Borderline BR (*n* = 7)
Age (years)	37 ± 15	46 ± 11
Gender (F/M)	15/14	1/6
BMI (kg/m^2^)	28 ± 6	30 ± 7
FEV1/FVC	0.83 + 0.06	0.8 ± 0.06
FEV_1_ (% of predicted)	97 ± 11 *	78 ± 13
FVC (% of predicted)	97 ± 14 *	80 ± 16
PeakVO_2_ (ml/kg/min)	30.6 ± 9.5	33.8 ± 12
PeakVO_2_ (%pred)	95 ± 19	105 ± 22
RER	1.19 ± 0.1	1.18 ± 0.16
Peak HR (bpm)	178 ± 17	169 ± 20
Peak HR (%pred)	99 ± 6.5	96 ± 10.6
VE/VCO_2_ slope	31 ± 7.5	30 ± 4.5
Peak O_2_ pulse (%pred)	93 ± 25 *	108 ± 13
SpO_2_ pretest (%)	97 ± 1.2	97 ± 5
Peak SpO_2_ (%)	98 ± 1.4	97 ± 5
VE (L/min)	101 ± 27	124 ± 38
MVV (L/min)	128 ± 35	110 ± 31
BR (L)	39 ± 18 *	4 ± 12
BR (%)	28 ± 9 *	3.7 ± 10

* Statistically significant between normal and low/borderline breathing reserve; CPET—cardiopulmonary exercise testing; BMI—body mass index; FEV_1_—forced expiratory volume in the first second; FVC—forced vital capacity; PeakVO_2_—peak oxygen uptake; RER—respiratory exchange ratio; HR—heart rate; VE/VCO_2_—minute ventilation/carbon dioxide production; SpO_2_—oxygen saturation; Peak SpO_2_—oxygen saturation at peak exercise; MVV—maximal voluntary ventilation; BR—breathing reserve.

## Data Availability

The data that support the findings of this study are available from the corresponding author Ronen Bar-Yoseph, upon reasonable request.

## Abbreviations

BR—Breathing reserve, CFS—chronic fatigue syndrome, COVID-19, coronavirus disease 2019, CPET—Cardio-pulmonary exercise test, DLCO—Diffusion capacity, FEV_1_—forced expiratory volume in the first second, FVC—forced vital capacity, PC19C—post-COVID-19 condition, PFT’s—Pulmonary function testing, SARS-CoV-2—severe acute respiratory syndrome coronavirus-2, TLC—total lung capacity, 6MWT—six-minute walking test.

## References

[1] Wu Z., McGoogan J.M. (2020). Characteristics of and Important Lessons from the Coronavirus Disease 2019 (COVID-19) Outbreak in China: Summary of a Report of 72314 Cases from the Chinese Center for Disease Control and Prevention. JAMA.

[2] Richardson S., Hirsch J.S., Narasimhan M., Crawford J.M., McGinn T., Davidson K.W., Barnaby D.P., Becker L.B., Chelico J.D., The Northwell COVID-19 Research Consortium (2020). Presenting Characteristics, Comorbidities, and Outcomes Among 5700 Patients Hospitalized With COVID-19 in the New York City Area. JAMA.

[3] Petrilli C.M., Jones S., Yang J., Rajagopalan H., O’Donnell L., Chernyak Y., Tobin K.A., Cerfolio R.J., Francois F., Horwitz L.I. (2020). Factors associated with hospital admission and critical illness among 5279 people with coronavirus disease 2019 in New York City: Prospective cohort study. BMJ.

[4] Yek C., Warner S., Wiltz J.L., Sun J., Adjei S., Mancera A., Silk B.J., Gundlapalli A.V., Harris A.M., Boehmer T.K. (2022). Risk Factors for Severe COVID-19 Outcomes Among Persons Aged ≥18 Years Who Completed a Primary COVID-19 Vaccination Series—465 Health Care Facilities, United States, December 2020–October 2021. Morb. Mortal. Wkly. Rep..

[5] Schaller T., Hirschbühl K., Burkhardt K., Braun G., Trepel M., Märkl B., Claus R. (2020). Postmortem Examination of Patients With COVID-19. JAMA.

[6] Ackermann M., Verleden S.E., Kuehnel M., Haverich A., Welte T., Laenger F., Vanstapel A., Werlein C., Stark H., Tzankov A. (2020). Pulmonary Vascular Endothelialitis, Thrombosis, and Angiogenesis in COVID-19. N. Engl. J. Med..

[7] Wichmann D., Sperhake J.P., Lütgehetmann M., Steurer S., Edler C., Heinemann A., Heinrich F., Mushumba H., Kniep I., Schröder A.S. (2020). Autopsy Findings and Venous Thromboembolism in Patients With COVID-19. Ann. Intern. Med..

[8] Grasselli G., Zangrillo A., Zanella A., Antonelli M., Cabrini L., Castelli A., Cereda D., Coluccello A., Foti G., Fumagalli R. (2020). Baseline Characteristics and Outcomes of 1591 Patients Infected With SARS-CoV-2 Admitted to ICUs of the Lombardy Region, Italy. JAMA.

[9] Wang Y., Dong C., Hu Y., Li C., Ren Q., Zhang X., Shi H., Zhou M. (2020). Temporal changes of CT findings in 90 patients with COVID-19 pneumonia: A longitudinal study. Radiology.

[10] Hui D.S., Joynt G.M., Wong K.T., Gomersall C.D., Li T.S., Antonio G., Ko F.W., Chan M.C., Chan D.P., Tong M.W. (2005). Impact of severe acute respiratory syndrome (SARS) on pulmonary function, functional capacity and quality of life in a cohort of survivors. Thorax.

[11] Hui D.S., Wong K.T., Ko F.W., Tam L.S., Chan D.P., Woo J., Sung J.J. (2005). The 1-year impact of severe acute respiratory syndrome on pulmonary function, exercise capacity, and quality of life in a cohort of survivors. Chest.

[12] Ngai J.C., Ko F.W., Ng S.S., To K.W., Tong M., Hui D.S. (2010). The long-term impact of severe acute respiratory syndrome on pulmonary function, exercise capacity and health status. Respirology.

[13] Park W.B., Jun K.I., Kim G., Choi J.P., Rhee J.Y., Cheon S., Lee C.H., Park J.S., Kim Y., Joh J.S. (2018). Correlation between pneumonia severity and pulmonary complications in Middle East respiratory syndrome. J. Korean Med. Sci..

[14] Lam M.H., Wing Y., Yu M.W., Leung C.M., Ma R.C., Kong A.P., So W.Y., Fong S.Y., Lam S.P. (2009). Mental Morbidities and Chronic Fatigue in Severe Acute Respiratory Syndrome Survivors: Long-term Follow-up. Arch. Intern. Med..

[15] George P.M., Wells A.U., Jenkins R.G. (2020). Pulmonary fibrosis and COVID-19: The potential role for antifibrotic therapy. Lancet Respir. Med..

[16] Tenforde M.W., Kim S.S., Lindsell C.J., Billig Rose E., Shapiro N.I., Files D.C., Gibbs K.W., Erickson H.L., Steingrub J.S., Smithline H.A. (2020). Symptom Duration and Risk Factors for Delayed Return to Usual Health Among Outpatients with COVID-19 in a Multistate Health Care Systems Network—United States, March–June 2020. Morb. Mortal. Wkly. Rep..

[17] Nalbandian A., Sehgal K., Gupta A., Madhavan M.V., McGroder C., Stevens J.S., Cook J.R., Nordvig A.S., Shalev D., Sehrawat T.S. (2021). Post-acute COVID-19 syndrome. Nat. Med..

[18] Driggin E., Madhavan M.V., Bikdeli B., Chuich T., Laracy J., Biondi-Zoccai G., Brown T.S., Der Nigoghossian C., Zidar D.A., Haythe J. (2020). Cardiovascular Considerations for Patients, Health Care Workers, and Health Systems during the COVID-19 Pandemic. J. Am. Coll. Cardiol..

[19] Gao Y., Chen R., Geng Q., Mo X., Zhan C., Jian W., Li S., Zheng J. (2021). Cardiopulmonary Exercise Test might be Helpful for Insight Interpretation of Impaired Pulmonary Function on Recovered COVID-19 Patients. Eur. Respir. J..

[20] Raveendran A.V., Jayadevan R., Sashidharan S. (2021). Long COVID: An overview. Diabetes Metab. Syndr..

[21] Mohr A., Dannerbeck L., Lange T.J., Pfeifer M., Blaas S., Salzberger B., Hitzenbichler F., Koch M. (2021). Cardiopulmonary exercise pattern in patients with persistent dyspnoea after recovery from COVID-19. Multidiscip. Respir. Med..

[22] Clavario P., De Marzo V., Lotti R., Barbara C., Porcile A., Russo C., Beccaria F., Bonavia M., Bottaro L.C., Caltabellotta M. (2021). Cardiopulmonary exercise testing in COVID-19 patients at 3 months follow-up. Int. J. Cardiol..

[23] Alba G.A., Ziehr D.R., Rouvina J.N., Hariri L.P., Knipe R.S., Medoff B.D., Hibbert K.A., Kowal A., Hoenstine C., Ginns L.C. (2021). Exercise performance in patients with post-acute sequelae of SARS-CoV-2 infection compared to patients with unexplained dyspnea. EClinicalMedicine.

[24] Barbagelata L., Masson W., Iglesias D., Lillo E., Migone J.F., Orazi M.L., Maritano Furcada J. (2021). Cardiopulmonary Exercise Testing in Patients with Post-COVID-19 Syndrome. Med. Clin..

[25] Cassar M.P., Tunnicliffe E.M., Petousi N., Lewandowski A.J., Xie C., Mahmod M., Samat A.H.A., Evans R.A., Brightling C.E., Ho L.P. (2021). Symptom Persistence Despite Improvement in Cardiopulmonary Health—Insights from longitudinal CMR, CPET and lung function testing post-COVID-19. EClinicalMedicine.

[26] Clinical Management of COVID-19: Interim Guidance, 27 May 2020. World Health Organization. https://apps.who.int/iris/handle/10665/332196.

[27] Culver B.H., Graham B.L., Coates A.L., Wanger J., Berry C.E., Clarke P.K., Hallstrand T.S., Hankinson J.L., Kaminsky D.A., MacIntyre N.R. (2017). ATS Committee on Proficiency Standards for Pulmonary Function Laboratories. Recommendations for a Standardized Pulmonary Function Report. An Official American Thoracic Society Technical Statement. Am. J. Respir. Crit. Care Med..

[28] Quanjer P.H., Stanojevic S., Cole T.J., Baur X., Hall G.L., Culver B.H., Enright P.L., Hankinson J.L., Ip M.S.M., Zheng J. (2012). ERS Global Lung Function Initiative. Multi-ethnic reference values for spirometry for the 3–95 yr age range: The global lung function 2012 equations. Eur. Respir. J..

[29] Lang R.M., Badano L.P., Mor-Avi V., Afilalo J., Armstrong A., Ernande L., Flachskampf F.A., Foster E., Goldstein S.A., Kuznetsova T. (2015). Recommendations for cardiac chamber quantification by echocardiography in adults: An update from the American Society of Echocardiography and the European Association of Cardiovascular Imaging. J. Am. Soc. Echocardiogr..

[30] ATS Committee on Proficiency Standards for Clinical Pulmonary Function Laboratories (2002). ATS statement: Guidelines for the six-minute walk test. Am. J. Respir. Crit. Care Med..

[31] Ross R.M. (2003). ATS/ACCP statement on cardiopulmonary exercise testing. Am. J. Respir. Crit. Care Med..

[32] Tanaka H., Monahan K.D., Seals D.R. (2001). Age-predicted maximal heart rate revisited. J. Am. Coll. Cardiol..

[33] Wasserman K., Hansen J.E., Sue D.Y., Stringer W.W., Sietsema K.E., Sun X.G., Whipp B.J. (2012). Principles of Exercise Testing and Interpretation.

[34] Huang Y., Tan C., Wu J., Chen M., Wang Z., Luo L., Zhou X., Liu X., Huang X., Yuan S. (2020). Impact of coronavirus disease 2019 on pulmonary function in early convalescence phase. Respir. Res..

[35] Xiong Q., Xu M., Li J., Liu Y., Zhang J., Xu Y., Dong W. (2021). Clinical sequelae of COVID-19 survivors in Wuhan, China: A single-centre longitudinal study. Clin. Microbiol. Infect..

[36] Halpin S.J., McIvor C., Whyatt G., Adams A., Harvey O., McLean L., Walshaw C., Kemp S., Corrado J., Singh R. (2021). Postdischarge symptoms and rehabilitation needs in survivors of COVID-19 infection: A cross-sectional evaluation. J. Med. Virol..

[37] Cares-Marambio K., Montenegro-Jiménez Y., Torres-Castro R., Vera-Uribe R., Torralba Y., Alsina-Restoy X., Vasconcello-Castillo L., Vilaró J. (2021). Prevalence of potential respiratory symptoms in survivors of hospital admission after coronavirus disease 2019 (COVID-19): A systematic review and meta-analysis. Chronic Respir. Dis..

[38] Huang L., Yao Q., Gu X., Wang Q., Ren L., Wang Y., Hu P., Guo L., Liu M., Xu J. (2021). 1-year outcomes in hospital survivors with COVID-19: A longitudinal cohort study. Lancet.

[39] Abdallah S.J., Voduc N., Corrales-Medina V.F., McGuinty M., Pratt A., Chopra A., Law A., Garuba H.A., Thavorn K., Reid R.E.R. (2021). Symptoms, Pulmonary Function, and Functional Capacity Four Months after COVID-19. Ann. Am. Thorac. Soc..

[40] Porfidia A., Valeriani E., Pola R., Porreca E., Rutjes A.W.S., Di Nisio M. (2020). Venous thromboembolism in patients with COVID-19: Systematic review and meta-analysis. Thromb. Res..

[41] Kollias A., Kyriakoulis K.G., Lagou S., Kontopantelis E., Stergiou G.S., Syrigos K. (2021). Venous thromboembolism in COVID-19: A systematic review and meta-analysis. Vasc. Med..

[42] Puntmann V.O., Carerj M.L., Wieters I., Fahim M., Arendt C., Hoffmann J., Shchendrygina A., Escher F., Vasa-Nicotera M., Zeiher A.M. (2020). Outcomes of Cardiovascular Magnetic Resonance Imaging in Patients Recently Recovered from Coronavirus Disease 2019 (COVID-19). JAMA Cardiol..

[43] Kravchenko D., Isaak A., Zimmer S., Mesropyan N., Reinert M., Faron A., Pieper C.C., Heine A., Velten M., Nattermann J. (2021). Cardiac MRI in Patients with Prolonged Cardiorespiratory Symptoms after Mild to Moderate COVID-19. Radiology.

[44] Mantovani E., Mariotto S., Gabbiani D., Dorelli G., Bozzetti S., Federico A., Zanzoni S., Girelli D., Crisafulli E., Ferrari S. (2021). Chronic fatigue syndrome: An emerging sequela in COVID-19 survivors?. J. Neurovirol..

[45] Khamsi R. (2021). Rogue antibodies could be driving severe COVID-19. Nature.

[46] Lui D.T.W., Lee C.H., Chow W.S., Lee A.C.H., Tam A.R., Fong C.H.Y., Law C.Y., Leung E.K.H., To K.K.W., Tan K.C.B. (2021). Thyroid dysfunction in relation to immune profile, disease status, and outcome in 191 patients with COVID-19. J. Clin. Endocrinol. Metab..

[47] Bornstein S.R., Rubin F., Ludwig B., Rietzsch H., Schwarz P.E.H., Rodionov R.N., Khunti K., Hopkins D., Birkenfeld A.L., Boehm B. (2021). Consequences of the COVID-19 pandemic on patients with metabolic diseases. Nat. Metab..

[48] Bornstein S.R., Voit-Bak K., Donate T., Rodionov R.N., Gainetdinov R.R., Tselmin S., Kanczkowski W., Müller G.M., Achleitner M., Wang J. (2021). Chronic post-COVID-19 syndrome and chronic fatigue syndrome: Is there a role for extracorporeal apheresis?. Mol. Psychiatry.

[49] Singh I., Joseph P., Heerdt P.M., Cullinan M., Lutchmansingh D.D., Gulati M., Possick J.D., Systrom D.M., Waxman A.B. (2022). Persistent Exertional Intolerance After COVID-19: Insights from Invasive Cardiopulmonary Exercise Testing. Chest.

